# Prevalence and risk factors for transactional sex among Swedish-born and foreign-born MSM in Sweden

**DOI:** 10.1186/s12889-022-14764-8

**Published:** 2022-12-22

**Authors:** Sara Causevic, Mariano Salazar, Anna Mia Ekström, Torsten Berglund, Kristina Ingemarsdotter Persson, Mikael Jonsson, Jonas Jonsson, Susanne Strömdahl

**Affiliations:** 1 Global and Sexual Health (GloSH) Research Group, Department of Global Public Health, Karolinska Institutet, Widerströmska Huset, Tomtebodavägen 18A, 171 77 Stockholm, Sweden; 2 Department of Infectious Diseases, South Central Hospital, Stockholm, Sweden; 3 The Swedish Federation for Lesbian, Gay, Bisexual, Transgender and Queer Rights (Riksförbundet För Homosexuellas, Bisexuellas, Transpersoners, Queeras Och Intersexpersoners Rättigheter, RFSL), Stockholm, Sweden; 4 Department of Medical Sciences, Infectious Medicine, Uppsala University, Uppsala, Sweden

**Keywords:** Men who have sex with men, Sweden, Migrant, Transactional sex

## Abstract

**Background:**

Little is known about transactional sex (TS) (selling and buying sex) among men who have sex with men (MSM) in Sweden, especially among foreign-born MSM. This study aims to assess the prevalence and risk factors of TS (ever and in the previous five years) among MSM living in Sweden and to determine if there is a difference between Swedish-born MSM and foreign-born MSM.

**Methods:**

Swedish data from a multicountry online banner survey (EMIS-2017) was used (*n* = 4443). Multivariable regression analysis was applied to analyse the data.

**Results:**

The prevalence of ever-selling sex among all MSM participants was 13.2% and 5.9% in the previous five years. Selling sex ever and in the previous five years was higher among foreign-born MSM (16% and 8.4%, respectively) than Swedish-born MSM (12.7% and 5.4%, respectively). Among all participants, younger age (aOR:3.19, 95% CI:1.57–6.45) and really struggling to live on current income (aOR:3.37, 95% CI:2.29–4.96) increased the odds of selling sex. Being foreign-born MSM (aOR:1.33, 95% CI:1.02–1.73) and having had sex with a woman in the previous 12 months increased the odds of selling sex (aOR:1.44, 95% CI:1.00–2.07).

The prevalence of ever buying sex among MSM participants in Sweden was 10.8% and 6.7% in the previous five years, with the same trend among foreign-born MSM (11.6% and 6.9%, respectively) and Swedish-born MSM (10.7% and 6.6%, respectively). Higher education and not having a current partner increased the odds of buying sex. Younger age was protective for buying sex (aOR:0.05, 95% CI:0.02–0.14). Among the foreign-born MSM, the length of stay in Sweden decreased the odds of buying sex (aOR: 0.98, 95% CI: 0.96–0.99).

**Conclusions:**

The comparatively high prevalence of TS among MSM participants in Sweden, where buying sex is illegal, with a higher prevalence among foreign-born MSM participants, calls for sexual and reproductive health and rights interventions in this population. Increased attention, including HIV prevention programming and education, should be aimed at younger MSM, MSM struggling with their current income, and foreign-born MSM, as they are more likely to report selling sex.

## Background

Transactional sex (TS) is broadly defined as an exchange of money, goods, and services for sex, where the payment route is described as either buying or selling sex [1]. The prevalence of TS varies depending on age, gender, and sexual orientation, as well as socioeconomic status [1–3]. For example, a systematic review of the prevalence among young people found that TS among boys occurred to a greater extent than among girls in high-income countries, with the reverse trend in low and middle-income countries [3]. The review indicated that TS prevalence rates among young people vary from below 10% in high-income countries to 5–85% among countries in sub-Saharan Africa [3]. A recent study from Sweden reported that 1.1% of 3256 school students engaged in TS in 2020, of which 1.4% were girls and 0.8% were boys [4].

In general, people engage in TS for different reasons. Studies among men who have sex with men (MSM) around the world have found that MSM sell sex to cover their basic needs (e.g., food, shelter, transportation), fund alcohol or drug use, improve their social status, feel empowered and seek adventure but also engage in TS as an expected part of courtship between partners [1, 5–8]. However, data highlights a power dimension of TS in that socioeconomic status influences the direction of TS relationships, and as a result, sellers have an increased risk of violence exposure [2, 9]. Lastly, buying sex can be more frequent among those with higher socioeconomic status, such as travellers who buy sex abroad in economically-deprived countries [2, 10].

Transactional sex is a key determinant of physical, mental, and sexual health among MSM. Different studies have reported that MSM who sell sex have a higher risk of acquiring a sexually transmitted infection (STI), experiencing various forms of violence (emotional, physical, sexual), abusing substances, using condoms inconsistently, and failing to disclose their HIV status to clients [1, 6, 11–13]. There is also a bidirectional association between mental health and sexual risk-taking behaviour, where they influence each other and increase the odds of poor mental and sexual health outcomes [14, 15].

The migration process has been associated with a higher risk of engaging in TS through different pathways [2, 16]. For example, migrants experiencing hardship with unsatisfied basic needs, such as lack of food and shelter in the receiving country (including undocumented migrants), may resort to TS to fulfil these needs. In doing so, they may be subjected to TS in the form of sexual violence [16–18]. Newly arrived migrants often lack a protective social network, miss their family and community, are more likely to be financially vulnerable, and may be unaware of their legal rights, which can further increase their risk of engaging in TS [19]. Data from the 2010 European MSM Internet Survey (EMIS–2010) showed that selling sex was more common among those born outside their current country of residence [2]. Lastly, the stigma against a same-sex relationship and strong gender norms combined with migration status and trajectory can impact risk behaviors such as engaging in buying sex and condomless sex as an expression of masculinity [20–22].

The International Organization for Migration (IOM) defines a migrant as “any person changing their country of residence, including asylum seekers, undocumented, labour and family migrants, students, researchers, and (unaccompanied) children under 18 years, who have moved from their usual place of residence, either in-country or across the border, for various time and reasons” [23]. In Sweden, almost 20% of the residents are foreign-born [24]. One reason for migration to Sweden includes homophobia or discrimination due to belonging to a sexual minority in the country of origin [25]. Sweden has comparatively liberal views towards different sexual orientations, ranking 9^th^ at the European level on lesbian, gay, bisexual, trans, queer, and intersex (LGBTQI) human rights [26]. However, a 2021 scoping review of violence and abuse against sexual and gender minority migrants revealed that violence and abuse occur throughout the migration trajectory, including in the destination country, where they may experience stigma and discrimination by the host and diaspora communities [27].

Little is known about the prevalence and the underlying causes of engaging in TS among MSM in Sweden. Swedish law prohibits purchasing sexual services, making it difficult to document this practice [28]. Our study aims to contribute to filling this knowledge gap by measuring the prevalence of TS (buying and selling sex) among MSM living in Sweden who participated in the European Men who have Sex with Men Internet Survey from 2017 (EMIS–2017). In addition, we assess if being a foreign-born MSM increases the odds of selling and buying sex and if there is any association between socioeconomic status, reasons for moving to Sweden, and length of stay in Sweden.

## Methods

### Study design, data collection and population

This paper used secondary data collected with the EMIS-2017 [29]. EMIS-2017 is an anonymous, cross-sectional, open-access, self-completion online survey that collected key health and demographic information about the MSM population accross 50 predominantly European countries from October 2017–January 2018 [29]. The sampling procedures and sample calculation are described elsewhere [29]. Participants who reported living in Sweden (n = 4443) were eligible for this study.

### Study variables

#### Dependent variables

Selling and buying sex refers to exchanging money, gifts, or favors for sex [1]. We estimated the prevalence of ever-selling and buying sex, and selling and buying sex during the previous five years. These time frames were chosen because very few informants reported selling or buying sex in the previous year (3.2% and 4.0%, respectively). By “previous five years”, we mean from the time when the survey was conducted (2017).

Two binary variables (*Yes/No*) were created to measure the “Ever” and “Previous five years” prevalence of selling sex using the question, *“When was the last time you were paid by a man to have sex with him? By paid, we mean he gave you money, gifts, or favours in return for sex*.” The selling sex (ever) variable was created with a binary outcome categorized as *Yes* (engaging in selling sex at any point of their life) and *No* (those who answered they have never sold sex). We categorized the selling sex in the previous five years variable as *Yes* (engaged in the practice within the previous 5 years) and *No* (those who never engaged in the practice). We took the same approach for the buying sex variable, created by asking, “*When was the last time you paid a man to have sex with you? By paid, we mean you gave him money, gifts, or favours in return for sex*.”

#### Independent variables

##### Demographic variables

We included the following variables: age in years (< *20, 20–24, 25–29, 30–34, 35–39, 40–44, 45–49, 50–54, 55–59, 60–64,* and ≥ *65)*, education, categorized as *low-level, mid-level (at least upper secondary;2–5 years spent in full-time education since the age of 16 years)* and *high-level (first stage of tertiary education or more)*, and current partnership status (having a steady partner)*,* categorized as *No (I am single), not sure,* or *Yes, I have a steady partner.* Socioeconomic status was assessed using a proxy question inquiring about current financial coping (*living really comfortably, living comfortably, neither comfortable nor struggling, struggling,* or *really struggling).*

##### Migration-related variables

We measured three variables: country of birth was used as a proxy measure for migration (*Swedish-born* or *foreign-born)*; length of stay in Sweden (measured as *years);* and reasons for migration (*came as a refugee/asylum seeker, came to live more openly as gay/bisexual/trans*, or *other).*

##### Gender and outness-related variables

We measured gender identity (*man* or *trans man*) and sexual orientation (*gay or homosexual*, *bisexual, straight or heterosexual, any other term, or I do not usually use a term).* Outness was defined as the proportion of people who knew about the respondents' attraction to men (*all or almost all, more than half, less, few,* or *none).*

##### Sexual risk-taking variables

Two variables were measured: 1. the number of sex partners in the previous 12 months (*no sex partners, one sex partner, 2–5 partners, 6–10 partners,* or *more than 10 partners)*, and 2. having sex with a woman in the previous 12 months (*No* or *Yes*).

##### Injecting drug use

We categorized the times of injection drug use (other than anabolic steroids or medicines) in the previous 12 months as *Never,* or *Once and more than once*.

### Data analysis

We conducted a complete case analysis since our data presented different patterns of missingness. In the overall dataset, demographic variables were 83% complete. The most common variables with missing data were education (12%) and outness (3%). The same pattern was observed among the Swedish-born MSM population. In addition, once we stratified by country of birth, in the foreign-born MSM population, we also observed a similar pattern of missingness among the variables education (9%), outness (2%), and length of stay in Sweden (2%).

Univariate and bivariate analyses are presented as percentages, means, and standard deviations. Pearson chi-square and t-tests were used to compare differences between groups. For simplicity, we only report the comparison between never and ever buying sex/ selling sex in the bivariate analysis.

For the multivariable analysis, we report the results of statistical models of both selling and buying sex (ever and in the previous five years) for all participants and stratified by country or birth (Swedish- and foreign-born). We used multivariable logistic regression to estimate adjusted odds ratios (aOR) and 95% confidence intervals (CI) between the dependent and independent variables. We included variables of interest shown to influence selling or buying sex in the analysis [1, 8, 30–33]. In addition, we included the reason for migration and the length of stay in Sweden in the foreign-born MSM analyses. All analyses were considered statistically significant if *p* < 0.05.

The presence of multicollinearity was assessed by calculating the variance inflation factor (VIF) and tolerance values of the independent variables through linear regression. Tolerance values less than 0.10 and VIF values above 10 were considered to signal collinearity [34]. The tolerance values ranged from 0.59 to 0.97, and VIF values ranged from 1.19–1.25, indicating no collinearity between the variables in our data.

For selling sex, we fitted three models for ever-selling sex in all MSM participants (Model 1), Swedish-born MSM (Model 2), and foreign-born MSM (Model 3). We fitted separate models for selling sex in the previous five years in all MSM participants (Model 4), Swedish-born MSM (Model 5), and foreign-born MSM (Model 6).

We fitted separate models of ever-buying sex among all MSM participants (Model 7), Swedish-born MSM (Model 8), and foreign-born MSM (Model 9). We also fitted separate models for buying sex in the previous five years in all MSM (Model 10), Swedish-born MSM (Model 11,) and foreign-born MSM (Model 12).

Data were analysed using Stata v16 (StataCorp, College Station, Texas).

## Results

### Descriptive analysis

#### All MSM participants

As presented in Table 1, the overall study population comprised 4443 participants with a median age of 46 years old, and 42.8% of the participants were older than 50 years. Participants were mainly born in Sweden (85.2%), and almost half reported having at least upper secondary education (47.2%). Most identified as men (97.2%), and 63.4% identified as gay or homosexual. The percentage of outness was 45.1%, and 56.4% did not have a current partner. A third (35.3%) reported living comfortably on their present income. The majority (80.1%) have not had sex with a woman in the past 12 months (Table 1). Most (97.7%, *n* = 4415) had never used injectable drugs.

**Table 1:** Descriptive and bivariate analysis of demographic characteristics and selected variables for all MSM, stratified by the selling and buying sex outcome variable (*n* = 4443)

Characteristics	All MSM n (%)	Selling sex		Buying sex	
		**Never n (%)**	**Ever n (%)**	**Never n (%)**	**Ever n (%)**
**Age**		***p* < *0.05***		***p* < *0.05***	
<20	111 (2.5)	57 (1.5)	34 (5.8)	91 (2.4)	0 (0.0)
20–24	290 (6.5)	219 (5.9)	57 (9.7)	271 (7.1)	5 (1.04)
25–29	426 (9.6)	308 (8.3)	101 (17.2)	398 (10.4)	9 (1.9)
30–34	392 (8.8)	312 (8.4)	68 (11.6)	363 (9.5)	18 (3.7)
35–39	419 (9.4)	338 (9.1)	67 (11.4)	367 (9.6)	38 (7.9)
40–44	452 (10.2)	382 (10.3)	62 (10.5)	396 (10.4)	47 (9.8)
45–49	450 (10.1)	398 (10.7)	45 (7.6)	375 (9.8)	66 (13.7)
50–54	554 (12.5)	490 (13.2)	50 (8.5)	480 (12.6)	58 (12.1)
55–59	476 (10.7)	441 (11.9)	26 (4.4)	397 (10.4)	70 (14.5)
60–64	363 (8.2)	324 (8.7)	30 (5.1)	291 (7.6)	62 (12.9)
≥ 65	510 (11.5)	447 (12.0)	48 (8.2)	388 (10.2)	108 (22.4)
*Total*	*4443 (100)*	*3716 (100)*	*588 (100)*	*3817 (100)*	*481 (100)*
**Country of birth**		***p* < *0.05***		*p* > *0.05*	
Foreign-born	656 (14.8)	524 (14.1)	105 (17.9)	555 (14.6)	76 (15.9)
Swedish-born	3775 (85.2)	3183 (85.9)	481 (82.1)	3253 (85.4)	403 (84.1)
*Total*	*4431 (100)*	*3707 (100)*	*586 (100)*	*3808 (100)*	*479 (100)*
**Education**		***p* < *0.05***		***p* < *0.05***	
Low level	270 (7.0)	220 (6.8)	32 (6.5)	227 (6.8)	23 (5.6)
Mid-level (upper secondary at least; 2–5 years spent in full-time education since the age of 16 years)	1818 (47.2)	1511 (46.4)	262 (53.1)	1603 (48.1)	166 (40.7)
High-level (first stage of tertiary education or more)	1764 (45.8)	1524 (46.8)	199 (40.4)	1502 (45.1)	219 (53.7)
*Total*	*3852 (100)*	*3255 (100)*	*493 (100)*	*3332 (100)*	*408 (100)*
**Current gender identity**		*p* > *0.05*		***p* < *0.05***	
Man	4321 (97.2)	3622 (97.5)	565 (96.1)	3705 (97.1)	476 (99.0)
Trans man	122 (2.8)	94 (2.5)	23 (3.9)	112 (2.9)	5 (1.0)
*Total*	*4443 (100)*	*3716 (100)*	*588 (100)*	*3817 (100)*	*481 (100)*
**Sexual orientation**		***p* < *0.05***		***p* < *0.05***	
Gay or homosexual	2814 (63.4)	2335 (62.9)	408 (69.5)	2380 (62.4)	359 (74.8)
Bisexual	1210 (27.3)	1045 (28.2)	120 (20.4)	1070 (28.1)	94 (19.6)
Straight or heterosexual	118 (2.7)	103 (2.8)	8 (1.4)	103 (2.7)	8 (1.7)
Any other term	96 (2.2)	75 (2.0)	16 (2.7)	85 (2.2)	5 (1.0)
I do not usually use a term	199 (4.5)	153 (4.1)	35 (6.0)	174 (4.6)	14 (2.9)
*Total*	*4437 (100)*	*3711 (100)*	*587 (100)*	*3812 (100)*	*480 (100)*
**People who knew about attraction to men (Outness)**		***p* < *0.05***		***p* < *0.05***	
All or almost all	1935 (45.1)	1588 (44.4)	312 (54.1)	1648 (44.8)	249 (53.8)
More than half	551 (12.9)	448 (12.5)	89 (15.4)	470 (12.8)	66 (14.2)
Less than half	376 (8.8)	317 (8.9)	46 (8.0)	328 (8.9)	34 (7.3)
Few	878 (20.5)	750 (21.0)	89 (15.4)	754 (20.5)	84 (18.1)
None	548 (12.8)	470 (13.2)	41 (7.1)	481 (13.1)	30 (6.5)
*Total*	*4288 (100)*	*3573 (100)*	*577 (100)*	*3681 (100)*	*463 (100)*
**Having a steady partner (current partner)**		***p* < *0.05***		*p* > *0.05*	
No, and not sure	2505 (56.4)	2050 (55.2)	363 (61.7)	2133 (56.0)	279 (58.1)
Yes, I have a steady partner	1933 (43.6)	1661 (44.8)	225 (38.3)	1680 (44.0)	201 (41.9)
*Total*	4438 (100)	*3711 (100)*	*588 (100)*	*3813 (100)*	*480 (100)*
**Number of sex partners in the previous 12 months**		***p* < *0.05***		***p* < *0.05***	
No sex partners	663 (14.9)	513 (13.8)	50 (8.5)	512 (13.4)	50 (10.4)
One partner	880 (19.8)	790 (21.3)	67 (11.4)	805 (21.1)	49 (10.2)
2–5 partners	1445 (32.6)	1275 (34.3)	159 (27.0)	1294 (33.9)	139 (28.9)
6–10 partners	651 (14.7)	554 (14.9)	96 (16.3)	569 (15.0)	81 (16.8)
More than 10 partners	801 (18.0)	584 (15.7)	216 (36.7)	637 (16.7)	162 (33.7)
*Total*	*4440 (100)*	*3716 (100)*	*588 (100)*	*3817 (100)*	*481 (100)*
**Sex with a woman in the previous 12 months**		*p* > *0.05*		***p* < *0.05***	
No	3537 (80.1)	2950 (79.8)	483 (82.3)	3013 (79.3)	415 (86.6)
Yes	881 (19.9)	748 (20.2)	104 (17.7)	787 (20.7)	64 (13.4)
*Total*	*4418 (100)*	*3698 (100)*	*587 (100)*	*3800 (100)*	*479 (100)*
**Living on present income**		***p* < *0.05***		*p* > *0.05*	
Living really comfortably	1208 (23.3)	892 (24.1)	107 (18.3)	868 (22.8)	130 (27.1)
Living comfortably	1560 (35.3)	1354 (36.6)	161 (27.5)	1340 (35.3)	174 (36.2)
Neither comfortable nor struggling	1097 (24.8)	914 (24.7)	145 (24.8)	958 (25.2)	99 (20.3)
Struggling	454 (10.3)	354 (9.6)	91 (15.6)	400 (10.5)	44 (9.2)
Really struggling	283 (6.4)	188 (5.1)	81 (13.8)	235 (6.2)	33 (6.9)
*Total*	*4422 (100)*	*3702 (100)*	*585 (100)*	*3801 (100)*	*480 (100)*

*n*=number of observations

#### Swedish-born MSM participants

The demographics of the 3775 Swedish-born MSM participants similarly reflected all MSM participants, where 44.9% of study participants were 50 years and older, and almost half had at least upper secondary education (50.6%) (Table 2).

**Table 2:** Descriptive and bivariate analysis of demographic characteristics and selected variables, stratified by selling and buying sex (*n* = 3775 Swedish-born MSM, *n* = 656 foreign-born MSM)

Characteristics	Swedish-born MSM n (%)	Foreign-born MSM n (%)	Selling sex				Buying sex			
			**Never n (%)**	**Ever n (%)**	**Never n (%)**	**Ever n (%)**	**Never n (%)**	**Ever n (%)**	**Never n (%)**	**Ever n (%)**
			**Swedish-born MSM**		**Foreign-born MSM**		**Swedish-born MSM**		**Foreign-born MSM**	
**Age**			***p* < *0.05***		***p* < *0.05***		***p* < *0.05***		***p* < *0.05***	
<20	101 (2.7)	10 (1.5)	53 (64.6)	29 (35.4)	4 (44.4)	5 (55.6)	82 (100.0)	0 (0.0)	9 (100.0)	0 (0.0)
20–24	238 (6.3)	52 (7.9)	180 (79.3)	47 (20.7)	39 (79.6)	10 (20.4)	225 (99.1)	2 (0.9)	46 (93.9)	3 (6.1)
25–29	353 (9.4)	73 (11.1)	256 (75.7)	82 (24.3)	52 (73.2)	19 (26.8)	330 (98.2)	6 (1.8)	68 (95.8)	3 (4.2)
30–34	290 (7.7)	102 (15.6)	234 (82.7)	49 (17.3)	78 (80.4)	19 (19.6)	274 (96.8)	9 (3.2)	89 (90.8)	9 (9.2)
35–39	342 (9.1)	77 (11.7)	281 (84.9)	50 (15.1)	57 (77.0)	17 (23.0)	307 (92.7)	24 (7.3)	60 (81.1)	14 (18.9)
40–44	375 (9.9)	75 (11.4)	323 (87.5)	46 (12.5)	58 (79.4)	15 (20.6)	330 (89.7)	38 (10.3)	65 (89.0)	8 (11.0)
45–49	382 (10.1)	68 (10.4)	338 (89.9)	38 (10.1)	60 (89.5)	7 (10.5)	316 (84.5)	58 (15.5)	59 (88.1)	8 (11.9)
50–54	501 (13.3)	51 (7.8)	446 (90.6)	46 (9.3)	44 (93.6)	3 (6.4)	437 (89.2)	53 (10.8)	42 (89.4)	5 (10.6)
55–59	419 (11.1)	53 (8.1)	389 (94.4)	23 (5.6)	48 (94.1)	3 (5.9)	354 (85.9)	58 (14.1)	40 (78.4)	11 (21.6)
60–64	324 (8.6)	38 (5.8)	288 (91.1)	28 (8.9)	35 (94.6)	2 (5.4)	258 (81.9)	57 (18.1)	32 (86.5)	5 (13.5)
≥ 65	450 (11.9)	57 (8.7)	395 (90.2)	43 (9.8)	49 (90.7)	5 (9.3)	340 (77.6)	98 (22.4)	45 (81.8)	10 (12.0)
*Total*	*3775 (100)*	*656 (100)*	*3183 (86.9)*	*481 (13.1)*	*524 (83.3)*	*105 (16.7)*	*3253 (88.9)*	*403 (11.0)*	*555 (88.0)*	*76 (12.0)*
**Country of birth**										
Foreign-born	*-*	656 (100)	*-*	*-*	*-*	*-*	*-*	*-*	*-*	*-*
Sweden	3775 (100)	*-*	*-*	*-*	*-*	*-*	*-*	*-*	*-*	*-*
*Total*	*3775 (100)*	*656 (100)*	*-*	*-*	*-*	*-*	*-*	*-*	*-*	*-*
**Education**			***p* < *0.05***		*p* > *0.05*		*p* > *0.05*		*p* > *0.05*	
Low level	235 (7.2)	34 (5.8)	196 (89.1)	24 (10.9)	23 (74.2)	8 (25.8)	197 (90.4)	21 (9.6)	29 (93.5)	2 (6.5)
Mid-level (upper secondary at least; 2–5 years spent in full-time education since the age of 16 years)	1647 (50.6)	168 (28.6)	1375 (85.5)	233 (15.5)	135 (82.8)	28 (17.2)	1451 (90.5)	152 (9.5)	150 (91.5)	14 (8.5)
High-level (first stage of tertiary education or more)	1373 (42.2)	385 (65.6)	1203 (89.5)	141 (10.5)	315 (84.5)	58 (15.5)	1179 (87.8)	163 (12.2)	318 (85.2)	55 (14.8)
*Total*	*3255 (100)*	*587 (100)*	*2774 (87.5)*	*398 (12.5)*	*473 (83.4)*	*94 (16.6)*	*2827 (89.4)*	*336 (10.6)*	*497 (87.5)*	*71 (12.5)*
**Migration due to homophobia**					*p* > *0.05*				*p* > *0.05*	
Refugee and asylum seekers	-	96 (14.6)	-	-	73 (88.9)	13 (15.1)	-	-	75 (87.2)	11 (12.8)
Migrated to live more openly as a gay/bisexual/trans	-	75 (11.4)	-	-	56 (76.7)	17 (23.3)	-	-	62 (84.9)	11 (15.1)
Other	-	482 (73.5)	-	-	395 (84.2)	75 (15.8)	-	-	417 (88.7)	53 (11.3)
*Total*	-	*653 (100)*	-	-	*524 (83.3)*	*105 (16.7)*	-	-	*554 (88.0)*	*75 (12.0)*
**Current gender identity**			***p* < *0.05***		*p* > *0.05*		***p* < *0.05***		*p* > *0.05*	
Man	3668 (97.2)	641 (97.7)	3102 (87.1)	459 (12.9)	511 (83.1)	104 (16.9)	3154 (88.8)	399 (11.2)	542 (87.8)	75 (12.2)
Trans man	107 (2.8)	15 (2.3)	81 (78.6)	22 (21.4)	13 (92.9)	1 (7.1)	99 (96.1)	4 (3.9)	13 (92.9)	1 (7.1)
*Total*	*3775 (100)*	*656 (100)*	*3183 (86.9)*	*481 (13.1)*	*524 (83.3)*	*105 (16.7)*	*3253 (89.0)*	*403 (11.0)*	*555 (88.0)*	*76 (12.0)*
**Sexual orientation**			***p* < *0.05***		*p* > *0.05*		***p* < *0.05***		*p* > *0.05*	
Gay or homosexual	2357 (62.5)	454 (69.5)	1964 (85.3)	338 (14.7)	369 (84.3)	69 (15.7)	1991 (86.7)	306 (13.3)	387 (88.1)	52 (11.9)
Bisexual	1078 (28.6)	126 (19.3)	941 (90.5)	99 (9.5)	99 (82.5)	21 (17.5)	958 (92.3)	80 (7.7)	108 (89.3)	13 (10.7)
Straight or heterosexual	105 (2.8)	13 (2.0)	93 (93.0)	7 (7.0)	10 (90.9)	1 (9.1)	94 (94.0)	6 (6.0)	9 (81.9)	2 (18.2)
Any other term	76 (2.0)	20 (3.1)	60 (83.3)	12 (16.7)	15 (78.9)	4 (21.1)	69 (97.2)	2 (2.8)	16 (84.2)	3 (15.8)
I do not usually use a term	157 (4.2)	40 (6.1)	123 (83.1)	25 (16.9)	28 (73.7)	10 (26.3)	139 (93.9)	9 (6.1)	33 (86.8)	5 (13.2)
*Total*	*3773 (100)*	*653 (100)*	*3181 (86.9)*	*481 (13.1)*	*521 (83.2)*	*105 (16.7)*	*3251 (89.0)*	*403 (11.0)*	*553 (88.1)*	*75 (11.9)*
**People who knew about attraction to men (Outness)**			***p* < *0.05***		*p* > *0.05*		***p* < *0.05***		*p* > *0.05*	
All or almost all	1642 (45.1)	292 (46.0)	1352 (83.7)	263 (16.3)	236 (83.1)	48 (16.9)	1398 (86.8)	213 (13.2)	250 (87.7)	35 (12.3)
More than half	451 (12.4)	99 (15.6)	371 (84.7)	67 (15.3)	76 (77.5)	22 (22.5)	386 (88.3)	51 (11.7)	83 (84.7)	15 (15.3)
Less than half	309 (8.5)	67 (10.5)	260 (87.2)	38 (12.8)	57 (87.7)	8 (12.3)	266 (89.6)	31 (10.4)	62 (95.4)	3 (4.6)
Few	751 (20.6)	123 (19.3)	649 (90.1)	71 (9.9)	97 (84.3)	18 (15.7)	646 (90.0)	72 (10.0)	105 (90.5)	11 (9.5)
None	488 (13.4)	55 (9.3)	427 (92.8)	33 (7.2)	39 (83.0)	8 (17.0)	435 (94.6)	25 (5.4)	42 (89.4)	5 (10.6)
*Total*	*3641 (100)*	*636 (100)*	*3059 (86.6)*	*472 (13.4)*	*505 (82.9)*	*104 (17.1)*	*3131 (88.9)*	*392 (11.1)*	*542 (88.7)*	*69 (11.3)*
**Having a steady partner (current partner)**			***p* < *0.05***		*p* > *0.05*		*p* > *0.05*		*p* > *0.05*	
No, and not sure	2122 (56.3)	375 (57.3)	1752 (85.6)	295 (14.4)	292 (81.6)	66 (18.4)	1807 (88.4)	237 (11.6)	320 (88.9)	40 (11.1)
Yes, I have a steady partner	1650 (43.7)	279 (42.7)	1428 (88.5)	186 (11.5)	230 (86.5)	39 (14.5)	1443 (89.7)	166 (10.3)	234 (87.0)	35 (13.0)
*Total*	*3772 (100)*	*654 (100)*	*3180 (86.9)*	*481 (13.1)*	*522 (83.2)*	*105 (16.8)*	*3250 (89.0)*	*403 (11)*	*554 (88.1)*	*75 (11.9)*
**Number of sex partners in the previous 12 months**			***p* < *0.05***		***p* < *0.05***		***p* < *0.05***		*p* > *0.05*	
No sex partners	573 (15.2)	86 (13.1)	450 (91.6)	41 (8.4)	61 (89.7)	7 (10.3)	445 (90.8)	45 (9.2)	64 (94.1)	4 (5.9)
One partner	764 (20.3)	112 (17.1)	686 (92.0)	60 (8.0)	101 (93.5)	7 (6.5)	704 (94.7)	39 (5.3)	98 (90.7)	10 (9.3)
2–5 partners	1238 (32.8)	204 (31.2)	1097 (89.1)	134 (10.9)	175 (87.5)	25 (12.5)	1112 (90.5)	116 (9.5)	179 (88.6)	23 (11.4)
6–10 partners	557 (14.8)	93 (14.2)	478 (86.0)	78 (14.0)	75 (80.6)	18 (19.4)	491 (88.3)	65 (11.7)	78 (83.9)	15 (16.1)
More than 10 partners	641 (17.0)	160 (24.4)	472 (73.8)	168 (25.2)	112 (70.0)	48 (30.0)	501 (78.4)	138 (21.6)	136 (85.0)	24 (15.0)
*Total*	*3773 (100)*	*655 (100)*	*3183 (86.9)*	*481 (13.1)*	*524 (83.3)*	*105 (16.7)*	*3253 (89.0)*	*403 (11.0)*	*555 (88.0)*	*76 (12.0)*
**Sex with a woman in previous 12 months**			***p* < *0.05***		*p* > *0.05*		***p* < *0.05***		*p* > *0.05*	
No	2972 (79.1)	557 (85.6)	2491 (86.2)	397 (13.8)	453 (84.4)	84 (15.6)	2530 (87.8)	351 (12.2)	477 (88.5)	62 (11.5)
Yes	783 (20.9)	94 (14.4)	677 (89.1)	83 (10.9)	68 (76.4)	21 (23.6)	709 (93.4)	50 (6.6)	75 (84.3)	14 (15.7)
*Total*	*3755 (100)*	*651 (100)*	*3168 (86.9)*	*480 (13.2)*	*521 (83.2)*	*105 (16.8)*	*3239 (89.0)*	*401 (11.0)*	*552 (87.9)*	*76 (12.1)*
**Living on present income**			***p* < *0.05***		*p* > *0.05*		*p* > *0.05*		*p* > *0.05*	
Living really comfortably	883 (23.5)	141 (21.7)	773 (89.7)	89 (10.3)	116 (87.2)	17 (12.8)	749 (87.1)	111 (12.9)	116 (86.6)	18 (13.4)
Living comfortably	1337 (35.6)	218 (33.5)	1170 (90.1)	129 (9.9)	180 (84.9)	32 (15.1)	1150 (88.6)	148 (11.4)	187 (88.2)	25 (11.8)
Neither comfortable nor struggling	913 (24.3)	182 (28.0)	763 (86.7)	117 (13.3)	149 (84.2)	28 (15.8)	801 (91.3)	76 (8.7)	155 (87.1)	23 (12.9)
Struggling	388 (10.3)	65 (10.0)	306 (80.3)	75 (19.7)	48 (76.2)	15 (23.8)	341 (89.7)	39 (10.3)	58 (92.1)	5 (7.9)
Really struggling	238 (6.3)	45 (6.9)	159 (69.7)	69 (30.3)	29 (70.7)	12 (29.3)	199 (87.7)	28 (12.3)	36 (87.8)	5 (12.2)
*Total*	*3759 (100)*	*651 (100)*	*3171 (86.9)*	*479 (13.1)*	*522 (83.4)*	*104 (16.6)*	*3240 (89)*	*402 (11.0)*	*552 (87.9)*	*76 (12.1)*

*n*=number of observations

#### Foreign-born MSM participants

The foreign-born MSM study population comprised 656 participants; almost a third (30%) were above 50 years old, more than half were highly educated (65.6%), and 97.7% identified as men. More than half (69.5%) identified as gay or homosexual, and 33.5% lived comfortably on their present income. Sixty percent were born in Europe, a majority in northwestern Europe, followed by Asia (16%) and South America (10%). The foreign-born MSM study population’s mean number of years living in Sweden was 22 (SD = 16). About 74% of respondents reported migrating to Sweden for reasons other than coming as refugees/asylum seekers or coming to live freely as gay/homosexual (Table 2).

### Bivariate analysis

#### Selling sex

##### All MSM participants

The reported prevalence of ever-selling sex by all MSM participants was 13.2% (*p* < 0.05) and 5.9% in the previous five years (*p* < 0.05). Ever-selling sex was associated with age, country of birth, education, sexual orientation, outness, current partner, number of sex partners in the previous 12 months, living on present income, and injecting drug use (Table 2, *p* < 0.05).

##### Swedish-born MSM participants

The prevalence of selling sex ever and in the previous five years was reported by 12.7% (*p* < 0.05) and 5.4% (*p* < 0.05) of the Swedish-born MSM, respectively. Ever-selling sex was associated with age, education, current gender identity, sexual orientation, outness, having a current partner, the number of partners in the previous 12 months, having had sex with a woman in the previous 12 months, living on present income, and injecting drug use (Table 2, *p* < 0.05).

##### Foreign-born MSM participants

The prevalence of selling sex ever and in the previous five years was reported by 16% (*p* < 0.05) and 8.4% (*p* < 0.05) of the foreign-born MSM, respectively. The analysis also showed that ever-selling sex was associated with age and the number of sex partners in the previous 12 months. (Table 2, *p* < 0.05). Lastly, selling sex was associated with injecting drug use.

#### Buying sex

##### All MSM participants

The prevalence of buying sex ever and in the previous five years was reported by 10.8% (*p* < 0.05) and 6.7% (*p* < 0.05) of all MSM, respectively. Bivariate analysis showed that ever-buying sex was associated with age, education, current gender identity, sexual orientation, outness, number of sexual partners in the previous 12 months, sex with women in the previous 12 months, and injecting drug use (Table 2, *p* < 0.05).

##### Swedish-born MSM participants

The prevalence of buying sex ever and in the previous five years was reported by 10.7% (p < 0.05) and 6.6% (*p* < 0.05) of the Swedish-born MSM, respectively. Bivariate analysis showed that ever-buying sex was associated with age, current gender identity, sexual orientation, outness, number of sexual partners in the previous 12 months, sex with women in the previous 12 months, and injecting drug use (Table 2, *p* < 0.05).

##### Foreign-born MSM participants

The prevalence of buying sex ever and in the previous five years was reported by 11.6% (*p* < 0.05) and 6.8% (*p* < 0.05) of the foreign-born MSM participants, respectively (*p* < 0.05). Bivariate analysis showed that ever-buying sex was associated with age (Table 2, *p* < 0.05).

## Multivariable analysis

Tables 3 and 4 show the estimates of the adjusted ORs and 95% CI obtained with a multivariable logistic regression model.

**Table 3:** Estimates of selling sex among EMIS–2017 MSM participants in Sweden (multivariable regression model)

Characteristics	Selling sex					
	**Ever selling sex**			**Selling sex in the previous 5 years**		
	**All MSM *(n* = *3596)***	**Swedish-born MSM *(n* = *3051)***	**Foreign-born MSM *(n* = *534)***	**All MSM *(n* = *3699)***	**Swedish-born MSM *(n* = *3134)***	**Foreign-born MSM *(n* = *382)***
	aOR (95% CI)					
***Age (ref:*** ≥ *65****)***	*1.00 (reference)*					
<20	3.19 (1.58–6.45)*	2.88 (1.34–6.18)*	6.81 (0.76–61.10)	15.08 (5.48–41.52)*	10.59 (8.19–29.50)*	41.88 (2.77–632.96)*
20–24	1.89 (1.15–3.11)*	1.77 (1.03–3.05)*	2.51 (0.53–11.80)	9.01 (3.67–22.12)*	7.91 (3.13–19.93)*	3.51 (0.55–22.13)
25–29	2.35 (1.50–3.68)*	2.19 (1.35–3.56)*	2.92 (0.67–12.73)	8.03 (3.35–19.21)*	6.73 (2.75–16.43)*	3.31 (0.57–19.13)
30–34	1.69 (1.05–2.70)*	1.57 (0.94–2.64)	2.10 (0.51–8.59)	2.60 (1.00–6.69)*	1.92 (0.68–5.38)	1.43 (0.26–7.84)
35–39	1.67 (1.04–2.66)*	1.50 (0.90–2.49)	2.19 (0.52–9.12)	3.68 (1.47–9.15)*	2.77 (1.06–7.26)*	2.20 (0.40–12.10)
40–44	1.33 (0.83–2.12)	1.19 (0.71–1.99)	2.17 (0.52–9.06)	2.27 (0.88–5.86)	1.62 (0.58–4.44)	1.96 (0.34–11.28)
45–49	0.98 (0.60–1.61)	0.96 (0.56–1.63)	1.18 (0.26–5.32)	0.64 (0.19–2.13)	0.75 (0.22–2.49)	(empty)
50–54	0.80 (0.50–1.31)	0.80 (0.47–1.33)	0.72 (0.12–4.07)	0.90 (0.30–2.63)	0.67 (0.21–2.11)	(empty)
55–59	0.54 (0.31–0.96)*	0.51 (0.27–0.93)*	0.85 (0.15–4.79)	0.13 (0.01–1.12)	0.14 (0.02–1.14)	(empty)
60–64	0.85 (0.48–1.49)	0.88 (0.48–1.59)	0.82 (0.12–5.61)	0.39 (0.08–1.97)	0.40 (0.08–2.00)	(empty)
***Country of birth (ref: Sweden)***	*1.00 (reference)*					
Foreign-born	1.33 (1.02–1.73)*	-	-	1.83 (1.25–2.66)*	-	-
***Sexual orientation*** ***(ref: Gay or homosexual)***	*1.00 (reference)*					
Bisexual	0.93 (0.65–1.31)	0.94 (0.64–1.39)	1.07 (0.45–2.57)	1.05 (0.62–1.74)	1.27 (0.72–2.23)	0.44 (0.11–1.61)
Straight or heterosexual	0.76 (0.27–2.12)	0.76 (0.24–2.36)	1.09 (0.08–14.98)	0.86 (0.22–3.30)	0.84 (0.17–4.20)	0.66 (0.03–11.98)
Any other term	0.63 (0.31–1.27)	0.63 (0.27–1.41)	0.63 (0.15–2.67)	0.35 (0.12–1.02)	0.38 (0.10–1.32)	0.22 (0.02–1.99)
I do not usually use a term	1.57 (0.98–2.51)	1.53 (0.88–2.65)	1.29 (0.48–3.46)	2.37 (1.30–4.29)*	2.86 (1.42–5.77)*	1.22 (0.34–4.29)
***Education (ref: Low level)***	*1.00 (reference)*					
Mid-level (at least upper secondary; 2–5 years spent in full-time education since the age of 16 years)	0.99 (0.64–1.51)	1.11 (0.68–1.80)	0.51 (0.17–1.49)	0.74 (0.39–1.40)	0.64 (0.31–1.31)	1.09 (0.20–5.72)
High-level (first stage tertiary education/more)	0.74 (0.47–1.15)	0.79 (0.48–1.32)	0.44 (0.15–1.26)	0.56 (0.28–1.09)	0.44 (0.20–0.94)*	0.92 (0.18–4.56)
***Living on present income (ref: Living really comfortably)***	*1.00 (reference)*					
Living comfortably	0.94 (0.70–1.26)	0.89 (0.64–1.22)	1.19 (0.56–2.52)	1.40 (0.81–2.40)	1.53 (0.79–2.95)	1.07 (0.38–3.02)
Neither comfortable nor struggling	1.06 (0.77–1.45)	1.00 (0.71–1.41)	1.45 (0.67–3.14)	1.51 (0.87–2.61)	1.84 (0.95–3.53)	0.90 (0.30–2.72)
Struggling	1.85 (1.29–2.64)*	1.79 (1.21–2.64)*	2.22 (0.85–5.79)	2.80 (1.54–5.08)*	2.81 (1.38–5.72)*	3.90 (1.12–13.53)*
Really struggling	3.37 (2.29–4.96)*	3.35 (2.19–5.12)*	4.01 (1.48–10.89)*	5.17 (2.79–9.56)*	5.87 (2.84–12.11)*	3.73 (0.97–14.33)
***Length of stay in Sweden***	*1.00 (reference)*					
	-	-	0.99 (0.96–1.01)	*-*	-	0.98 (0.94–1.01)
***Reason for migration (ref: Refugees and asylum seekers*)**	*1.00 (reference)*					
Migrated to live openly as a gay/bisexual/trans	-	-	1.75 (0.67–4.51)	*-*	-	2.25 (0.63–8.07)
Other	-	-	1.02 (0.46–2.24)	*-*	-	1.61 (0.52–4.89)
***People who knew about attraction to men (Outness) (ref: All or almost all)***	*1.00 (reference)*					
More than half	0.97 (0.72–1.30)	0.88 (0.63–1.23)	1.35 (0.69–2.61)	1.34 (0.89–2.02)	1.10 (0.67–1.78)	2.69 (1.12–6.40)*
Less than half	0.71 (0.50–1.03)	0.76 (0.51–1.16)	0.43 (0.16–1.16)	0.55 (0.29–1.01)	0.68 (0.35–1.33)	0.15 (0.02–1.10)
Few	0.50 (0.36–0.71)*	0.47 (0.32–0.69)*	0.54 (0.24–1.21)	0.51 (0.30–0.86)*	0.42 (0.22–0.76)*	0.90 (0.28–2.80)
None	0.40 (0.25–0.63)*	0.36 (0.22–0.60)*	0.58 (0.18–1.86)	0.40 (0.21–0.77)*	0.24 (0.11–0.52)*	1.97 (0.44–8.83)
**Having a steady partner (current partner) * (ref: Yes, I have a steady partner)***	*1.00 (reference)*					
No, and not sure	0.96 (0.77–1.18)	0.92 (0.73–1.16)	1.12 (0.66–1.91)	0.97 (0.69–1.36)	1.04 (0.70–1.53)	0.77 (0.36–1.62)
**Sex with a woman in the previous 12 months *(ref: No)***	*1.00 (reference)*					
Yes	1.44 (1.00–2.07)*	1.32 (0.88–1.98)	2.83 (1.12–7.11)*	2.22 (1.33–3.70)*	2.16 (1.21 -3.83)*	4.12 (1.08–15.68)*

Legend: *n*=number of observations

*aOR* Adjusted Odds Ratios, *CI* Confidence intervals

^*^p<0.05

**Table 4:** Estimates of buying sex among EMIS–2017 MSM participants in Sweden (multivariable regression model)

Characteristics	Buying sex					
	**Ever buying sex**			**Buying sex in the previous 5 years**		
	**All MSM *(n* = *3532)***	**Swedish-born MSM *(n* = *2993)***	**Foreign-born MSM *(n* = *522)***	**All MSM *(n* = *3631)***	**Swedish-born MSM *(n* = *3073)***	**Foreign-born MSM *(n* = *537)***
	AOR (95% CI)					
***Age (ref:*** ≥ *65****)***	*1.00 (reference)*					
< 20	(empty)	(empty)	(empty)	(empty)	(empty)	(empty)
20–24	0.05 (0.02–0.14)*	0.01 (0.01–0.10)*	0.30 (0.05–1.69)	0.13 (0.05–0.33)*	0.04 (0.01–0.27)*	0.60 (0.07–4.76)
25–29	0.06 (0.03–0.13)*	0.04 (0.01–0.10)*	0.18 (0.03–0.99)*	0.10 (0.04–0.25)*	0.07 (0.02–0.23)*	0.17 (0.02–1.52)
30–34	0.15 (0.08–0.27)*	0.11 (0.05–0.22)*	0.34 (0.08–1.41)	0.21 (0.10–0.43)*	0.16 (0.07–0.39)*	0.31 (0.05–2.05)
35–39	0.35 (0.22–0.54)*	0.24 (0.14–0.40)*	1.14 (0.30–4.25)	0.47 (0.27–0.82)*	0.39 (0.21–0.72)*	0.74 (0.12–4.41)
40–44	0.40 (0.26–0.61)*	0.37 (0.23–0.58)*	0.74 (0.18–2.97)	0.63 (0.37–1.04)	0.62 (0.36–1.05)	0.71 (0.11–4.64)
45–49	0.54 (0.36–0.80)*	0.54 (0.35–0.82)*	0.50 (0.11–2.16)	0.80 (0.49–1.29)	0.77 (0.46–1.29)	0.68 (0.10–4.44)
50–54	0.49 (0.33–0.72)*	0.44 (0.29–1.22)*	0.89 (0.20–3.85)	0.74 (0.46–1.18)	0.68 (0.41–1.11)	1.34 (0.20–8.79)
55–59	0.64 (0.43–0.94)*	0.54 (0.35–0.82)*	2.21 (0.58–8.44)	0.71 (0.43–1.18)	0.64 (0.38–1.09)	1.81 (0.27–11.86)
60–64	0.72 (0.48–1.09)	0.68 (0.44–1.06)	1.72 (0.39–7.47)	0.75 (0.43–1.28)	0.65 (0.37–1.16)	3.51 (0.52–23.43)
***Country of birth (ref: Sweden)***	*1.00 (reference)*					
Foreign-born	1.20 (0.89–1.63)	-	-	1.13 (0.78–1.64)	-	-
***Sexual orientation (ref: Gay or homosexual)***	*1.00 (reference)*					
Bise﻿xual	0.65 (0.44–0.96)*	0.65 (0.42–0.99)*	0.69 (0.23–2.05)	0.92 (0.58–1.47)	0.93 (0.56–1.53)	1.06 (0.29–3.87)
Straight or heterosexual	0.57 (0.16–2.00)	0.74 (0.21–2.62)	(empty)	0.31 (0.04–2.41)	0.40 (0.05–3.13)	(empty)
Any other term	0.32 (0.07–1.36)	0.21 (0.03–1.58)	0.77 (0.09–6.77)	0.60 (0.14–2.57)	0.41 (0.05–3.10)	1.50 (0.15–14.88)
I do not usually use a term	0.72 (0.38–1.35)	0.54 (0.24–1.21)	1.08 (0.34–3.44)	0.85 (0.39–1.83)	0.72 (0.28–1.85)	1.08 (0.24–4.74)
***Education (ref: Low level)***	*1.00 (reference)*					
Mid-level (at least upper secondary; 2–5 years spent in full-time education since the age of 16 years)	1.41 (0.86–2.30)	1.40 (0.83–2.35)	1.60 (0.32–7.90)	1.40 (0.75–2.62)	1.37 (0.71–2.65)	1.54 (0.17–13.74)
High-level (first stage tertiary education/more)	1.70 (1.04–2.78)*	1.60 (0.95–2.70)	2.08 (0.45–9.69)	1.89 (1.01–3.52)*	1.75 (0.90–3.38)	2.06 (0.24–17.02)
***Living on present income (ref: Living really comfortably)***	*1.00 (reference)*					
Living comfortably	0.83 (0.62–1.08)	0.80 (0.59–1.07)	0.82 (0.38–1.76)	0.67 (0.48–0.92)*	0.63 (0.45–0.90)*	0.82 (0.32–2.08)
Neither comfortable nor struggling	0.77 (0.55–1.06)	0.72 (0.49–1.04)	0.90 (0.41–2.01)	0.53 (0.35–0.80)*	0.51 (0.32–0.80)*	0.57 (0.20–1.63)
Struggling	1.22 (0.81–1.83)	1.30 (0.83–2.01)	0.91 (0.28–2.87)	0.72 (0.43–1.21)	0.72 (0.41–1.26)	0.74 (0.17–3.16)
Really struggling	1.35 (0.84–2.17)	1.39 (0.81–2.35)	1.32 (0.40–4.37)	1.03 (0.59–1.81)	0.99 (0.52–1.84)	1.62 (0.41–6.39)
***Length of stay in Sweden***	*1.00 (reference)*					
	-	-	0.98 (0.95–0.99)*	*-*	-	0.96 (0.93–0.99)*
***Reason for migration (ref: Refugees and asylum seekers*)**	*1.00 (reference)*					
Migrated to live openly as a gay/bisexual/trans	-	-	0.94 (0.31–2.80)	*-*	-	1.43 (0.36–5.60)
Other	-	-	0.86 (0.37–2.01)	*-*	-	1.16 (0.38–3.42)
**People who knew about attraction to men (Outness) *(ref: All or almost all)***	*1.00 (reference)*					
More than half	0.96 (0.68–1.35)	0.83 (0.56–1.22)	1.45 (0.66–3.14)	0.96 (0.64–1.46)	0.82 (0.50–1.32)	1.66 (0.64–4.28)
Less than half	0.69 (0.45–1.04)	0.69 (0.49–1.09)	0.44 (0.12–1.59)	0.63 (0.36–1.07)	0.68 (0.39–1.19)	0.19 (0.03–1.61)
Few	0.78 (0.55–1.10)	0.71 (0.48–1.05)	0.78 (0.31–1.96)	0.73 (0.47–1.12)	0.72 (0.45–1.16)	0.45 (0.13–1.54)
None	0.52 (0.31–0.87)*	0.43 (0.24–0.75)*	1.09 (0.30–3.98)	0.57 (0.31–1.04)	0.50 (0.25–0.96)*	0.91 (0.20–4.06)
**Having a steady partner (current partner) * (ref: Yes, I have a steady partner)***	*1.00 (reference)*					
No, and not sure	1.27 (1.00–1.59)*	1.36 (1.06–1.75)*	1.08 (0.61–2.02)	1.38 (1.04–1.83)*	1.44 (1.06–1.96)*	1.30 (0.60–2.79)
**Sex with a woman in the previous 12 months *(ref: No)***	*1.00 (reference)*					
Yes	1.18 (0.77–1.80)	1.18 (0.73–1.89)	1.62 (0.54–4.83)	1.18 (0.72–1.94)	1.08 (0.62–1.87)	3.21 (0.92–11.24)

Legend: *n*=number of observations

*AOR* Adjusted odds ratio, *CI* Confidence interval

^*^p<0.05

### Ever selling sex

#### All MSM participants (Table 3)

Factors increasing the odds of ever-selling sex: when compared to those aged above 65 years, almost all age categories until 39 years old had increased odds of having ever-sold sex with aORs ranging from 3.19 (95% CI: 1.58–6.45) for those less than 20 years old to 1.67 (95% CI: 1.04–2.66) for those aged 35–39 years. Those born elsewhere had increased odds of ever-selling sex (aOR:1.33, 95% CI: 1.02–1.73) compared to those born in Sweden. Compared to those living really comfortably with their current income, those struggling (aOR 1.85, 95% CI: 1.29–2.64) and really struggling (aOR 3.37, 95% CI: 2.29–4.96) also had increased odds of ever-selling sex. Having sex with a woman also increased the odds (aOR:1.44, 95% CI: 1.00–2.07) for ever-selling sex.

Factors decreasing the odds of ever-selling sex: Compared to those who responded that all or almost all people knew about their attraction to men (outness), those who responded that only a few (aOR: 0.50, 95% CI: 0.36–0.71) or no one knew (aOR:0.40, 95% CI: 0.25–0.63) had lower odds of ever-selling sex. In addition, being 55–59 decreased the odds of ever-selling sex (aOR 0.54, 95% CI: 0.31–0.96).

#### Swedish-born MSM participants (Table 3)

The factors increasing the odds of ever-selling sex among the Swedish-born MSM followed a similar pattern as those reported among all MSM sample (younger age and struggling to live on current income). The factors decreasing the odds of ever-selling sex followed the same pattern as those found among all MSM (older age and outness).

#### Foreign-born MSM participants (Table 3)

The factors increasing the odds of ever-selling sex among the foreign-born MSM followed the same patterns as those reported among the all MSM sample (really struggling to live on current income) with the addition of the variable of having had sex with a woman in the previous twelve months (aOR: 2.83, 95% CI: 1.12–7.11). There were no protective factors for ever-selling sex in this subpopulation.

### Selling sex in the previous 5 years

#### All MSM participants (Table 3)

Factors increasing the odds of selling sex: almost all age categories until 39 years of age had increased odds of selling sex in the previous five years, with aORs ranging from 15.08 (95% CI: 5.48–41.52) for those aged less than 20 years to 3.68 (95% CI: 1.47–9.15) for those aged 35–39 years, when compared to those aged above 65 years. Compared to those MSM born in Sweden, those foreign-born MSM participants had increased odds of selling sex in the 5 years (aOR:1.83, 95% CI: 1.25–2.66). Compared to those living comfortably on their current income, those struggling financially (aOR:2.80, 95% CI: 1.54–5.08) and really struggling (aOR: 5.17, 95% CI: 2.79–9.56) had increased odds of selling sex in the previous five years. Compared to those identifying as gay or homosexual, those not usually using a term for their sexual orientation had higher odds of selling sex in the previous five years (aOR:2.37, 95% CI:1.30–4.29). Having sex with a woman in the previous 12 months also increased the odds (aOR: 2.22, 95% CI: 1.33–3.70) of selling sex in the previous five years.

Factors decreasing the odds of selling sex in the previous five years: Compared to those who reported that all or almost all people knew about their attraction to men, those who reported that only a few people (aOR:0.51, 95% CI: 0.30–0.86) or none knew (aOR:0.40, 95% CI: 0.21–0.77) had decreased odds of selling sex in the previous five years.

#### Swedish-born MSM participants (Table 3)

Factors that increased the odds of selling sex in the previous five years among the Swedish-born MSM followed similar patterns as those reported among all MSM sample (younger age, sexual orientation, struggling on current living income, and having had sex with a woman in the previous 12 months). The factors decreasing the odds followed the same pattern as those found among all MSM sample (education and outness).

#### Foreign-born MSM participants (Table 3)

Among the foreign-born MSM, only younger age, struggling to live on their current income, and having had sex with a woman in the previous 12 months followed the pattern of all MSM participants. The level of outness was a risk factor among foreign-born MSM participants. Compared to those who reported that all or almost all people knew about their attraction to men, those who reported that more than half knew had increased odds of selling sex in the previous five years (aOR: 2.69, 95% CI: 1.12–6.40). No protective factors were found for this subpopulation.

### Ever buying sex

#### All MSM participants (Table 4)

Factors increasing the odds of ever buying sex: Compared to those reporting low educational levels, having first-stage tertiary education increased the odds of ever buying sex (aOR: 1.70, 95% CI: 1.04–2.78). Compared to those with a current partner, those with no current partner had higher odds of ever buying sex (aOR: 1.27, 95% CI: 1.00–1.59).

Factors decreasing the odds of ever buying sex: When compared to those older than 65 years, almost all age categories (except those aged 60–64 years) had decreased odds of ever buying sex with aOR ranging from 0.05 (95% CI: 0.02–0.14) for those aged 20–24 years or less to 0.64 (95% CI: 0.43–0.94) for those aged 55–59 years. Compared to those who identified as gay or homosexual, those who identified as bisexual 0.65 (95% CI 0.44–0.96) had decreased odds of ever buying sex. In addition, compared to those whom all or almost all people knew about their attraction to men, those who reported no-one knowing 0.52 (95% CI: 0.31–0.87) had decreased odds of ever buying sex.

#### Swedish-born MSM participants (Table 4)

In this sub-population, not having a current partner (aOR: 1.36, 95% CI: 1.05–1.75) increased the odds of ever buying sex. The factors decreasing the odds of ever buying sex among the Swedish-born MSM followed the same patterns reported among all MSM (younger age, outness, and sexual orientation).

#### Foreign-born MSM participants (Table 4)

None of the studied factors increased the odds of ever buying sex in this subpopulation. Compared to the findings from the all MSM sample, only age in those 25–29 years old (aOR:0.18, 95% CI:0.03–0.99) and the length of stay in Sweden decreased the odds of ever buying sex (aOR:0.98, 95% CI: 0.96–0.99).

### Buying sex in the previous 5 years

#### All MSM participants (Table 4)

Compared to those who currently have a partner, those with no partner had higher odds of buying sex in the previous five years (aOR: 1.38, 95% CI:1.04–1.83). Compared to those with low-level education, those highly educated also had higher odds of buying sex in the previous 5 years (aOR:1.89, 95% CI: 1.01–3.52).

In addition, when compared to those aged above 65 years, almost all age categories below 40 years old had significantly lower odds of buying sex with aORs ranging from 0.10 (95% CI: 0.04–0.25) for those aged 25–29 years to 0.47 (95% CI: 0.27–0.82) for those aged 35–39 years. Compared to those living really comfortably on their current income, those living comfortably (aOR: 0.67, 95% CI 0.48–0.92) and those neither comfortable nor struggling (aOR: 0.53, 95% CI: 0.35–0.80) had lower odds of buying sex in the previous five years.

#### Swedish-born MSM (Table 4)

The factors increasing the odds of buying sex in the previous five years among the Swedish-born MSM followed the same patterns as those reported among all MSM (not having a current partner).

The factors decreasing the odds of buying sex in the previous five years followed the same pattern as those found among the all MSM sample (age and income) with the addition of outness. Compared to those where all or almost all people knew about their attraction to men, those reporting low outness had 0.50 (95% CI: 0.25–0.96) decreased odds of having bought sex in the previous five years.

#### Foreign-born MSM (Table 4)

We found no significant determinant of buying sex in the previous five years among this study population. The length of stay in Sweden (aOR:0.96, 95% CI: 0.93–0.99) decreased the odds of buying sex in the previous five years.

## Discussion

Our findings show an overall prevalence of selling sex ever and in the previous five years of 13.2% and 5.9%, respectively, among MSM in Sweden participating in the EMIS–2017. The overall prevalence of buying sex ever and in the previous five years was 10.8% and 6.7%, respectively. The prevalence of selling sex was significantly higher among foreign-born MSM than in the Swedish-born MSM (16% versus 12.7%). Age, socioeconomic status, and level of outness were factors associated with TS engagement in MSM in Sweden.

Our findings on the prevalence of selling and buying sex are consistent with the research on MSM populations in high-income countries [1, 2, 8, 32]. Studies such as the EMIS-2010 observed a 12.2% prevalence in TS from the total sample from 38 European countries [1, 2], while studies on the overall MSM population in other high-income countries show a prevalence ranging from 4.5–7% for selling sex and 6–17.1% for buying sex [8, 32]. In a cross-sectional study among Australian gay and bisexual men sex workers and their clients, from 2009, almost 17% of the participants sold sex to another man, and 25% were clients buying sex from another man [8].

Being of younger age decreased the odds of buying sex. At the same time, being younger increased the odds of selling sex, as observed in previous research where buying sex increased and selling sex declined steadily with age [8]. One of the possible explanations for this result is that the younger MSM could be in higher demand for having sex due to physical appearance and attractiveness [2, 6]. Further, younger people generally have less economic power; thus, older men would be able to cater to the young men and buy sex with favours, social introduction, and gifts, including lodging [3, 35]. Another possible explanation is that the transaction acts as a catalyst where without the transaction component, the younger MSM would not have had sex with the older one [35].

These findings could suggest a power imbalance between the age groups. Such power imbalance has been found potentially harmful if the younger MSM do not have social support, increasing their vulnerability and impairing their ability to negotiate the rules of the sexual encounter [2, 8, 33, 35]. Age discrepancy has also been reported to affect condom use as the older partner may hold the upper hand in condom use negotiations [36]. Finally, sexual identity exploration among young people may partly explain the age discrepancy found, where a more senior partner could lead a younger MSM through this phase as a protective figure, benefactor, and mentor [6, 32].

MSM struggling to live on their current income reported higher odds of selling sex. This finding is in line with previous studies showing that economic vulnerability affects the choices of where, when, and with whom to have sex [8, 37, 38]. Our findings are consistent with that of Prestage et al., who conducted a survey among 2306 gay and bisexual men sex workers and their clients in Australia in 2009, where those who were paid for sex were less likely to be in full-time employment and selling sex could be used to complement their monthly earnings [8]. In addition, similar findings were confirmed among homeless youth in the USA who engaged in TS due to a financial struggle [38].

Further, the findings from studies in Europe and the USA are aligned with our results, revealing a pattern of power interplays between buyers and sellers of sex due to financial differences [2, 39]. Complementing our results, those living well were at decreased odds of buying sex in the previous five years compared to those living very well, which was confirmed in a study on EMIS–2010 data revealing that a higher proportion of buyers had steady employment [2]. In conclusion, data indicates that selling sex among MSM is motivated by financial scarcity and social vulnerability and usually represents sex work [2].

We observed a negative association between outness, where few or none of the closest friends or family knew about participants’ attraction to men, and both selling and buying sex among all MSM, including Swedish-born MSM populations. In addition, our data showed that those not identifying themselves as gays/homosexual had lower odds of having ever bought sex. High levels of outness have been described as a key factor facilitating MSM access to LGBTQI networks, HIV/STI prevention programs and HIV testing, and higher risk-taking behaviours such as drug use and engaging in sex parties [40, 41]. One possible explanation is that MSM with low levels of outness and those who do not identify themselves as gay/homosexuals might have limited access to LGBTQI social networks or venues where TS is more common, thus limiting their opportunities to engage in TS [42]. More qualitative research is needed to understand in-depth the pathways and contextual factors that could affect engagement in TS and further explain our findings.

Being foreign-born MSM increased the odds of selling sex compared to Swedish-born MSM, further strengthened by the finding that foreign-born MSM with a high level of outness were at higher odds of selling sex in the previous five years. Foreign-born MSM might be more adversely affected by socioeconomic opportunities than natives. As mentioned in previous research, low income and ethnicity amplify the engagement in TS among MSM, including MSM having sex with women, possibly revealing sex work [43]. A similar result was observed in a study among MSM in the USA, where MSM with concurrent partnerships, including sex with women, reported a higher prevalence of TS and could be engaged in sex work [44]. Lastly, previous research, such as a mixed-methods study from Peru, has identified that MSM sex workers with with both male and female clients are less likely to access HIV prevention and testing and are at higher risk for STIs than MSM who have sex with men only [31, 45].

Our findings on age, economic imbalance, and country of birth reveal a socioeconomic hierarchy in the patterns of TS engagement among MSM in Sweden. Those who were more educated among the overall MSM population and did not have a current partner were more likely to be involved in buying sex and less likely to have sold sex. Our results showed a similar trend among the Swedish MSM population. A study from Australia on gay and bisexual male sex workers and their clients reported that those more likely to buy sex were fully employed, not in a relationship, and had a university education [8]. Higher education is usually related to employment and higher socioeconomic status, which enables the financial power to buy sex [2, 46]. In addition, buying sex could occur abroad, including in countries that are less economically advantaged than Sweden [2, 46].

Years spent living in Sweden reduced the likelihood of buying sex in the foreign-born MSM population. One explanation could be that buying sex is illegal in Sweden, and individuals may become more aware of Swedish laws and regulations over time spent living in Sweden. In addition, with time spent in Sweden, people learn about and adapt to social norms. Lastly, selling sex was more common than buying sex among all respondents in Sweden, which could also be a consequence of the criminalization of purchasing sex services in Sweden [2, 28].

### Limitations

Our study has some limitations. First, all data is self-reported, and there could be a recall or social desirability bias. TS is stigmatized in Sweden and buying sex is illegal, which could be a reason for underreporting the practice [28]. Second, the survey was an online banner survey representing a rather large convenience sample of 4443 MSM, however findings can not be generalized to the Swedish MSM population. The median age of the participants was in the older age group implying that the sample's representativeness should be interpreted with caution. Third, the survey was broad covering several topics rather than focusing on TS. More specific data on TS event's frequency and timeline would be valuable in future research. Further, data on if the transaction occurred in Sweden or abroad, if it was consensual, commercial, or part of a love relationship dynamic would increase our understanding of TS. The survey did not target TS with women as clients but did not exclude them either.

Finally, the data analysed for this study is from 2017, before the COVID-19 pandemic. The socioeconomic consequences following the pandemic could also have exacerbated the engagement in TS, especially in selling sex.

An explanation for many identified limitations is that the survey was a broad public health survey with several aims not limited to our specific study purposes. Additional qualitative research could add to the evidence and knowledge established in this research, aimed explicitly toward populations we did not manage to capture and to address underlying causes of engagement in TS.

Lastly, we are trying to describe the myriad of factors and risks for engaging in TS among MSM; however, we are aware there are multidimensional motivations and reasons that we did not present. Further research can build on our research and expand it for higher accuracy.

## Conclusions

Our findings show show a high prevalence of selling and buying sex among MSM participants, with a higher prevalence among foreign-born MSM in Sweden. Younger MSM, foreign-born MSM and those struggling with their current income were more likely to report having sold sex. The financial component can impact their possibility of negotiating safe sex practices making them more vulnerable to STIs including HIV.

These findings highlight the need for sexual and reproductive health and rights interventions, education, HIV/STI prevention programming, and support for MSM engaged in TS. The diversity of MSM, range of sexual identities, and male sex work should be considered in TS and sex work support and prevention programming.

## Data Availability

For questions on data availability that support the findings of this study, EMIS–2017 coordinators should be contacted at coordinator@emis-project.eu. Data could be available from the authors upon reasonable request and with the permission of EMIS–2017 coordinators.

## Abbreviations

aOR: Adjusted odds ratio
CI: Confidence interval
EMIS: European MSM internet survey
HIV: Human immunodeficiency virus
IOM: International organization for migration
LGBTQI: Lesbian, gay, bisexual, trans, queer, and intersex
MSM: Men who have sex with men
STI: Sexually transmitted infection
TS: Transactional sex
